# Histone lysine crotonylation during acute kidney injury in mice

**DOI:** 10.1242/dmm.024455

**Published:** 2016-06-01

**Authors:** Olga Ruiz-Andres, Maria Dolores Sanchez-Niño, Pablo Cannata-Ortiz, Marta Ruiz-Ortega, Jesus Egido, Alberto Ortiz, Ana Belen Sanz

**Affiliations:** 1 Nephrology, IIS-Fundacion Jimenez Diaz, Madrid 28040, Spain; 2 School of Medicine, UAM, Madrid 28029, Spain; 3 REDinREN, Madrid 28040, Spain; 4 Pathology, IIS-Fundacion Jimenez Diaz, Madrid 28040, Spain; 5 School of Medicine, UAM, Madrid 28029, Spain; 6 IRSIN, Madrid 28003, Spain

**Keywords:** Acute kidney injury, Epigenetics, Histone, Inflammation, Tubular cell

## Abstract

Acute kidney injury (AKI) is a potentially lethal condition for which no therapy is available beyond replacement of renal function. Post-translational histone modifications modulate gene expression and kidney injury. Histone crotonylation is a recently described post-translational modification. We hypothesized that histone crotonylation might modulate kidney injury. Histone crotonylation was studied in cultured murine proximal tubular cells and in kidneys from mice with AKI induced by folic acid or cisplatin. Histone lysine crotonylation was observed in tubular cells from healthy murine and human kidney tissue. Kidney tissue histone crotonylation increased during AKI. This was reproduced by exposure to the protein TWEAK in cultured tubular cells. Specifically, ChIP-seq revealed enrichment of histone crotonylation at the genes encoding the mitochondrial biogenesis regulator PGC-1α and the sirtuin-3 decrotonylase in both TWEAK-stimulated tubular cells and in AKI kidney tissue. To assess the role of crotonylation in kidney injury, crotonate was used to increase histone crotonylation in cultured tubular cells or in the kidneys *in vivo*. Crotonate increased the expression of PGC-1α and sirtuin-3, and decreased CCL2 expression in cultured tubular cells and healthy kidneys. Systemic crotonate administration protected from experimental AKI, preventing the decrease in renal function and in kidney PGC-1α and sirtuin-3 levels as well as the increase in CCL2 expression. For the first time, we have identified factors such as cell stress and crotonate availability that increase histone crotonylation *in vivo*. Overall, increasing histone crotonylation might have a beneficial effect on AKI. This is the first observation of the *in vivo* potential of the therapeutic manipulation of histone crotonylation in a disease state.

## Abstract (precis)

**Summary:** We have assessed the effect of the epigenetic post-translational modification histone crotonylation during kidney injury *in vivo* and in cell culture, and the involvement of PGC-1α and SIRT3 in the process.

## INTRODUCTION

Post-translational modifications of proteins are involved in chronic kidney disease and cardiovascular disease (Gajjala et al., 2015). Indeed, mounting evidence suggests that histone post-translational modifications, such as methylation, acetylation or phosphorylation, play a key role in diverse biological processes, such as development, cell differentiation, cell death and inflammation (Berdasco and Esteller, 2010). Indeed, aberrant histone post-translational modification contributes to disease (Berdasco and Esteller, 2010). Histone post-translational modifications regulate chromatin-templated processes through two major mechanisms (Kouzarides, 2007): modulating chromatin packaging and regulating chromatin structure and function by recruiting binding proteins specific to the post-translational modification, which recognize modified histones. Alternatively, histone post-translational modifications can also inhibit the interaction of specific binders with chromatin. Recent studies have identified lysine crotonylation (Kcr) as a novel evolutionarily conserved histone post-translational modification that is present in several somatic tissues from adult mice (Tan et al., 2011). As recently described, histone crotonylation is mechanistically and functionally different from histone lysine acetylation (Tan et al., 2011; Sabari et al., 2015). Histone crotonylation was observed in kidney tissue, suggesting that it might play a role in epigenetic regulation of gene expression during kidney injury (Tan et al., 2011).

There is as yet very little knowledge about the regulation and function of histone crotonylation during tissue injury. Crotonate is a short-chain unsaturated carboxylic acid (CH_3_CH=CHCO_2_H) that increases histone crotonylation in cultured non-renal cells (Tan et al., 2011; Sabari et al., 2015), but its effect *in vivo* has not been addressed (Tan et al., 2011). Sirtuin-3 (SIRT3) is a histone deacetylase, also recently identified as a decrotonylase (Tan et al., 2011). SIRT3 is a member of the sirtuin family of NAD(+)-dependent deacetylases (Huang et al., 2010; Scher et al., 2007) that associates with chromatin to repress nearby genes (Shechter et al., 2007; Iwahara et al., 2012). SIRT3 is present both in mitochondria and nuclei (Scher et al., 2007; Nakamura et al., 2008; Sundaresan et al., 2008), and is expressed in kidneys and metabolically active tissues. Under physiological and stress conditions, SIRT3 and peroxisome-proliferator-activated receptor gamma coactivator-1α (PGC-1α) regulate the expression of each other (Shi et al., 2005; Palacios et al., 2009; Than et al., 2011; Giralt et al., 2011). PGC-1α regulates gluconeogenesis and mitochondrial biogenesis and respiration. SIRT3 is a mediator of PGC-1α effects on mitochondrial biogenesis (Kong et al., 2010). PGC-1α is downregulated during acute kidney injury (AKI) (Ruiz-Andres et al., 2015; Tran et al., 2011), a condition characterized by a sudden, potentially prolonged, reduction of the renal glomerular filtration rate causing azotemia. AKI is associated with high morbidity and mortality rates and there is no therapy to treat established AKI beyond replacement of kidney function (Berger and Moeller, 2014). Proximal tubules are rich in mitochondria and are key sites of injury during AKI. An improved understanding of pathogenic pathways involved in AKI might provide clues to design novel therapeutic approaches. Inflammation and cell death are key contributors to AKI (Linkermann et al., 2014; Garcia-Cenador et al., 2013). In this regard, the protein tumor necrosis factor (TNF)-like weak inducer of apoptosis (TWEAK) has recently been shown to be a key contributor to AKI and kidney injury in general (Ortiz et al., 2011; Sanz et al., 2011). TWEAK is a cytokine of the TNF superfamily that activates the Fn14 receptor, and has multiple actions on kidney cells. Thus, TWEAK promotes kidney inflammation by increasing chemokine secretion by renal cells and decreasing the expression of nephroprotective factors such as klotho, promotes tubular cell proliferation in a permissive environment, and induces mesangial and tubular cell apoptosis under proinflammatory conditions (Sanz et al., 2011). TWEAK decreases PGC-1α and target gene expression in tubular cells through nuclear factor κB (NFκB) activation and histone deacetylation (Ruiz-Andres et al., 2015). Given that the actions of TWEAK are mediated through the recruitment of signaling mechanisms that include NFκB activation and histone acetylation, we hypothesized that TWEAK might also modulate histone lysine crotonylation. Furthermore, because epigenetic changes are also observed in AKI, and histone deacetylase (HDAC) inhibitors might protect from kidney injury (Van Beneden et al., 2011, 2013), we further hypothesized that histone lysine crotonylation might be a contributor to and a therapeutic target in AKI.

We have now explored histone crotonylation regulation and function in cultured kidney tubular epithelial cells and during kidney injury *in vivo*. Specifically, we have observed that histone lysine crotonylation is increased during AKI and by inflammatory cytokines such as TWEAK in tubular cells. Crotonate increased histone lysine crotonylation and PGC-1α expression in cultured tubular cells and in the kidney *in vivo*, and protected from AKI.

## RESULTS

### Histone crotonylation is increased in kidney tubular cells during acute kidney injury

The histone crotonylation pattern during renal injury was explored in detail in an established mouse model of AKI induced by a folic acid overdose, and the results were confirmed in experimental cisplatin-induced AKI. In folic-acid-induced AKI, as in other experimental models and human AKI, loss of renal function, tubular cell injury and interstitial inflammation were observed (Sanz et al., 2008a).

**Fig. 1.**
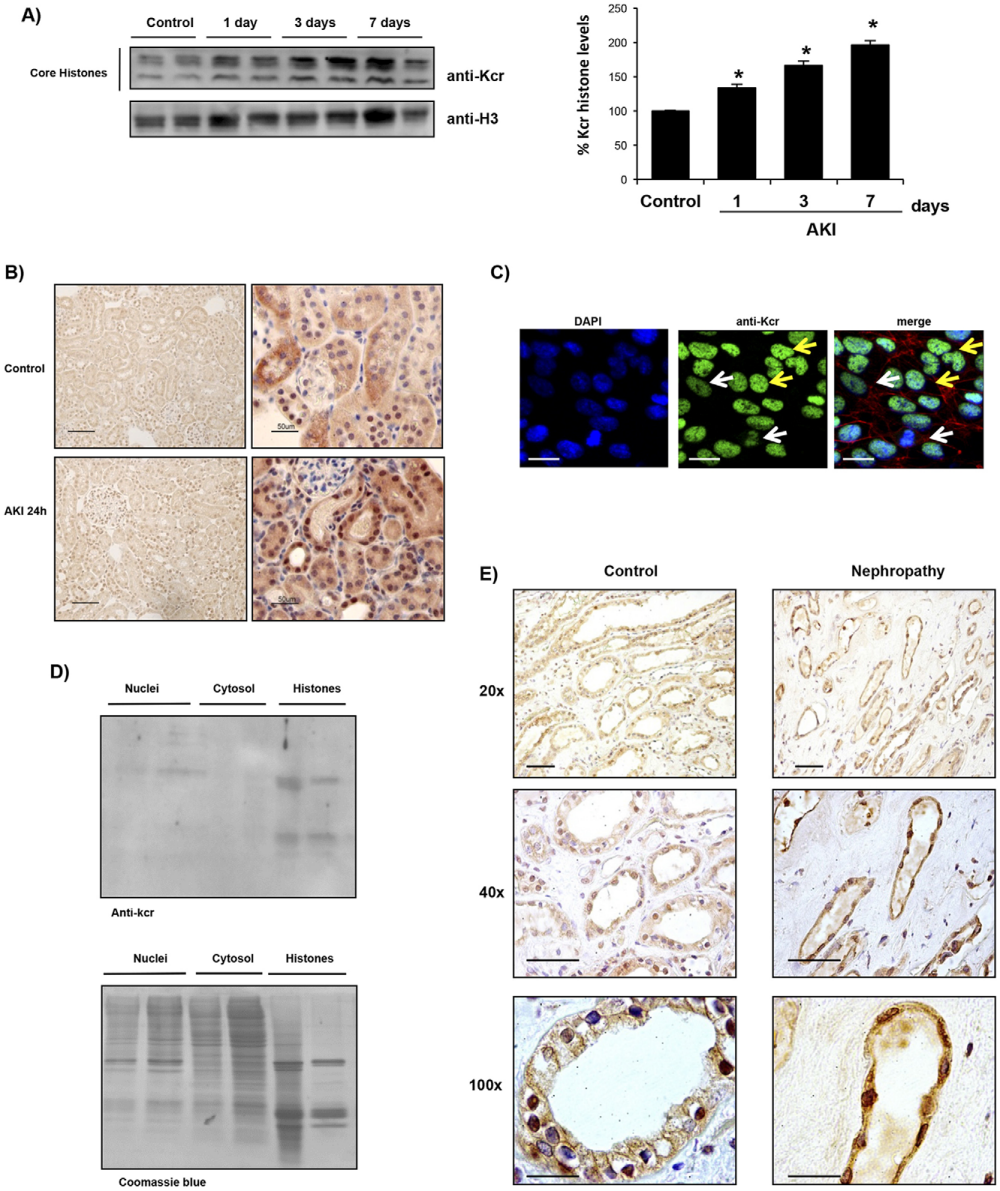
**Histone crotonylation in kidney tubular cells**. (A) Western blot of murine healthy control kidneys and animals subjected to acute kidney injury (AKI) induced by a folic acid overdose. A quantification expressed as the percentage change of crotonylated histones (anti-Kcr) over control is shown to the right (mean±s.e.m. of five animals per group). Histone-3 was used as a loading control. *P<0.05 vs control (nonparametric Mann–Whitney *U*-test). (B) Immunohistochemistry showing that histone crotonylation is mainly localized to tubular cell nuclei in murine kidneys. Representative staining of crotonylated histones (brown) counterstained with hematoxylin (blue). Scale bars: 100 µm (left-hand panels); 50 µm (right-hand panels). (C) Nuclear localization of histone crotonylation (green) was also observed in cultured murine proximal tubular cells. Nuclei were stained with DAPI (blue) and the actin cytoskeleton with fluorescent phalloidin (red). Note several degrees of histone crotonylation is observed; some cells have a lower staining intensity (white arrows) than others (yellow arrows). Scale bars: 5 µm. (D) Western blotting and Coomassie Blue stain of extracts from cultured tubular cells. There is a stronger Kcr signal in histones, corresponding to the faint bands in nuclei and the low cytosol signal. (E) Immunohistochemistry showing that histone crotonylation is also localized to tubular cells in human kidneys. Representative staining of histone crotonylation (brown) counterstained with hematoxylin (blue). Scale bars: 50 µm (20× panels); 50 µm (40× panels); 20 µm (100× panels).

Consistent with prior reports (Tan et al., 2011), histone crotonylation was observed in healthy murine kidney tissue when assessed by western blotting (Fig. 1A) or immunohistochemistry (Fig. 1B). Western blotting identified histones as crotonylated proteins, whereas immunohistochemistry localized lysine crotonylation mainly to tubular cell nuclei (Fig. 1B). Western blotting showed that there was an increase in the overall histone crotonylation in the folic-acid-induced AKI kidney tissue (Fig. 1A). Similar results were observed in cisplatin-induced AKI at 72 h (Fig. S1). The rest of the detailed studies discussed in the manuscript were obtained in the folic acid model. We also explored the histone H3 crotonylation (H3k9cr) pattern during renal injury. Consistent with the finding of overall histone crotonylation, western blotting showed that there was an increase in kidney histone H3 crotonylation in folic-acid-induced AKI tissue (Fig. S2A). Immunohistochemistry identified tubular cells as sites of lysine crotonylation during AKI (Fig. 1B). Nuclear localization of lysine crotonylation consistent with histone crotonylation was also observed in cultured murine proximal tubular cells by immunofluorescence (Fig. 1C). As was the case *in vivo*, there were different degrees of lysine crotonylation in individual cultured tubular cells, suggesting that this is a regulated and dynamic process. Cell separation into nuclei and cytosol showed that there was a faint crotonylated protein band in nuclei that corresponded to the size of histones (Fig. 1D). Thus, histones appear to be the most abundant crotonylated proteins in kidney tubular cells. Immunohistochemistry also identified nuclear lysine crotonylation in diseased human kidney tubular cells (Fig. 1E).

### TWEAK increases histone crotonylation in cultured kidney tubular cells

**Fig. 2.**
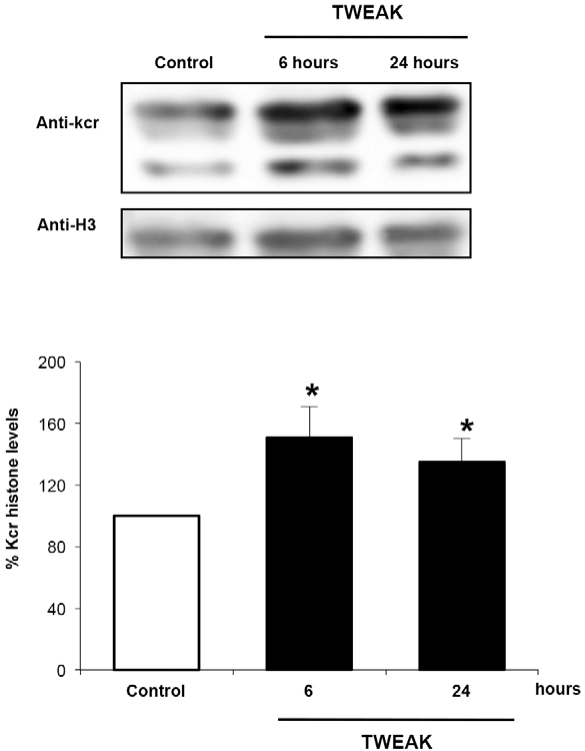
**TWEAK increases histone crotonylation in kidney tubular cells.** Western blotting of histone crotonylation (anti-Kcr) in cultured murine proximal tubular cells stimulated with 100 ng/ml TWEAK. Data from five independent experiments is expressed as the mean±s.e.m. Results are expressed as percentage change of crotonylated histones over control.*P<0.05 vs control (Student's *t*-test).

Next, we explored the hypothesis that inflammatory mediators of AKI could modulate histone crotonylation. TWEAK is a key mediator of AKI that promotes inflammatory responses in cultured tubular cells but has no direct cytotoxicity if used in the absence of other inflammatory mediators (Sanz et al., 2010b; Izquierdo et al., 2012). Hence, we studied the effect of TWEAK on histone crotonylation in kidney cells. TWEAK increased histone crotonylation at 6 and 24 h in cultured tubular cells (Fig. 2). These results suggest that inflammatory cytokines can regulate the histone crotonylation status in kidney cells.

At this point, we also explored the hypothesis that direct cytotoxicity might promote histone crotonylation. As is the case for folic-acid-induced AKI, ischemia-reperfusion-induced AKI and other forms of AKI, cisplatin promotes an inflammatory response that amplifies kidney injury *in vivo* (Zhang et al., 2008; Baek et al., 2015). However, in contrast to TWEAK, cisplatin has a direct toxic effect on cultured tubular cells. In this regard, cisplatin at concentrations that induced direct toxicity in cultured tubular cells, did not modify histone crotonylation (Fig. S3). These results indicate that cytotoxicity and histone crotonylation can be dissociated in cultured tubular cells and argue for the involvement of additional factors *in vivo*.

### Crotonate increases histone crotonylation and elicits biological responses in cultured tubular cells

**Fig. 3.**
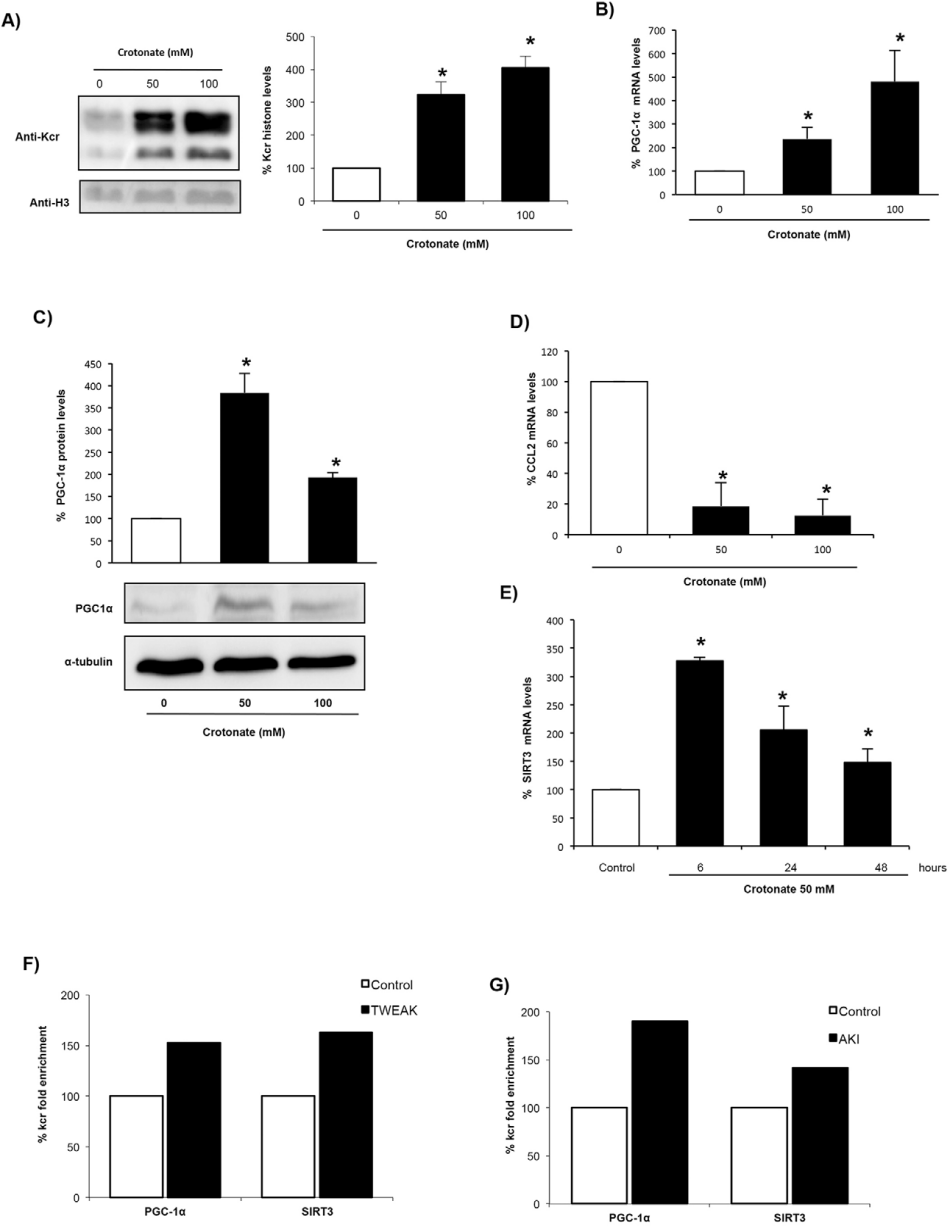
**Crotonate increases histone crotonylation and modifies gene expression in cultured proximal tubular epithelial cells.** Cells were stimulated with 0, 50 or 100 mM crotonate for 24 h. (A) Quantification of histone crotonylation (anti-Kcr) and representative western blot. Mean±s.e.m. of three independent experiments; *P<0.05 vs 0 mM crotonate (non-parametric Mann–Whitney *U*-test). (B,D) PGC-1α and CCL2 mRNA levels. Data from eight independent experiments expressed as mean±s.e.m. *P<0.05 vs control (Student's *t*-test). (C) PGC-1α western blot of whole-cell extracts. Data from four independent experiments is expressed as mean±s.e.m. *P<0.05 vs control (non-parametric Mann–Whitney *U*-test). (E) SIRT3 mRNA levels in tubular cells exposed to 50 mM crotonate. Data from four independent experiments expressed as mean±s.e.m. *P<0.05 vs control (non-parametric Mann–Whitney *U*-test). (F,G) ChIP-seq analysis was performed using a pan anti-Kcr antibody in (F) tubular cells incubated with 100 ng/ml TWEAK for 6 h (data from three independent experiments) and (G) kidney tissue from mouse AKI (six animals per group). Results show the percentage of Kcr enrichment.

Next, we searched for potential target genes of histone crotonylation whose expression is differentially regulated in AKI. PGC-1α is a regulator of mitochondrial biogenesis that is decreased in AKI, whereas SIRT3 is a decrotonylase, and both regulate the expression of each other. Therefore, as a representative downregulated gene we chose PGC-1α, because it regulates SIRT3 expression (Giralt et al., 2011; Kong et al., 2010; Bell and Guarente, 2011). Moreover, TWEAK decreases PGC-1α expression by epigenetic mechanisms involving histone acetylation (Ruiz-Andres et al., 2015). ChIP-seq analysis using the pan anti-crotonyl-lysine antibody showed that PGC-1α and SIRT3 were more enriched in crotonylated histones in tubular cells treated with TWEAK and in kidneys with AKI (Fig. 3F,G). To study the effect of crotonylation on PGC-1α and SIRT3 expression, cells were pretreated with crotonate because exogenous crotonate increased histone crotonylation in cultured tubular cells (Fig. 3A). This is consistent with findings in non-renal cells (Tan et al., 2011; Sabari et al., 2015). Crotonate increased tubular cell PGC-1α mRNA and protein levels (Fig. 3B,C). As representative upregulated gene, we chose CCL2 because it encodes the MCP-1 chemokine, a promoter of kidney injury (Sanz et al., 2010a). Crotonate decreased tubular cell CCL2 mRNA in cultured cells (Fig. 3D). Taken together, these results suggest that histone crotonylation could play an overall protective role in kidney injury by promoting upregulation of some protective genes and downregulation of genes involved in tissue injury. Crotonate also increased SIRT3 mRNA levels in cultured tubular cells in a time-dependent manner (Fig. 3E), suggesting the activation of a negative-feedback loop.

Crotonate did not promote tubular cell death nor proliferation as assessed by the presence of hypodiploid cells or by analyzing the proportion of cells in the S/M phases by flow cytometry (Fig. S4), nor did it increase cell detachment as observed by phase-contrast microscopy (data not shown). Mannitol, an osmolarity control, did not modify histone crotonylation at concentrations equimolar to the crotonate concentrations used, arguing against a role of osmolarity in modulating histone crotonylation (Fig. S5).

### Crotonate increases histone crotonylation and modulates regulated gene expression in mouse kidney

**Fig. 4.**
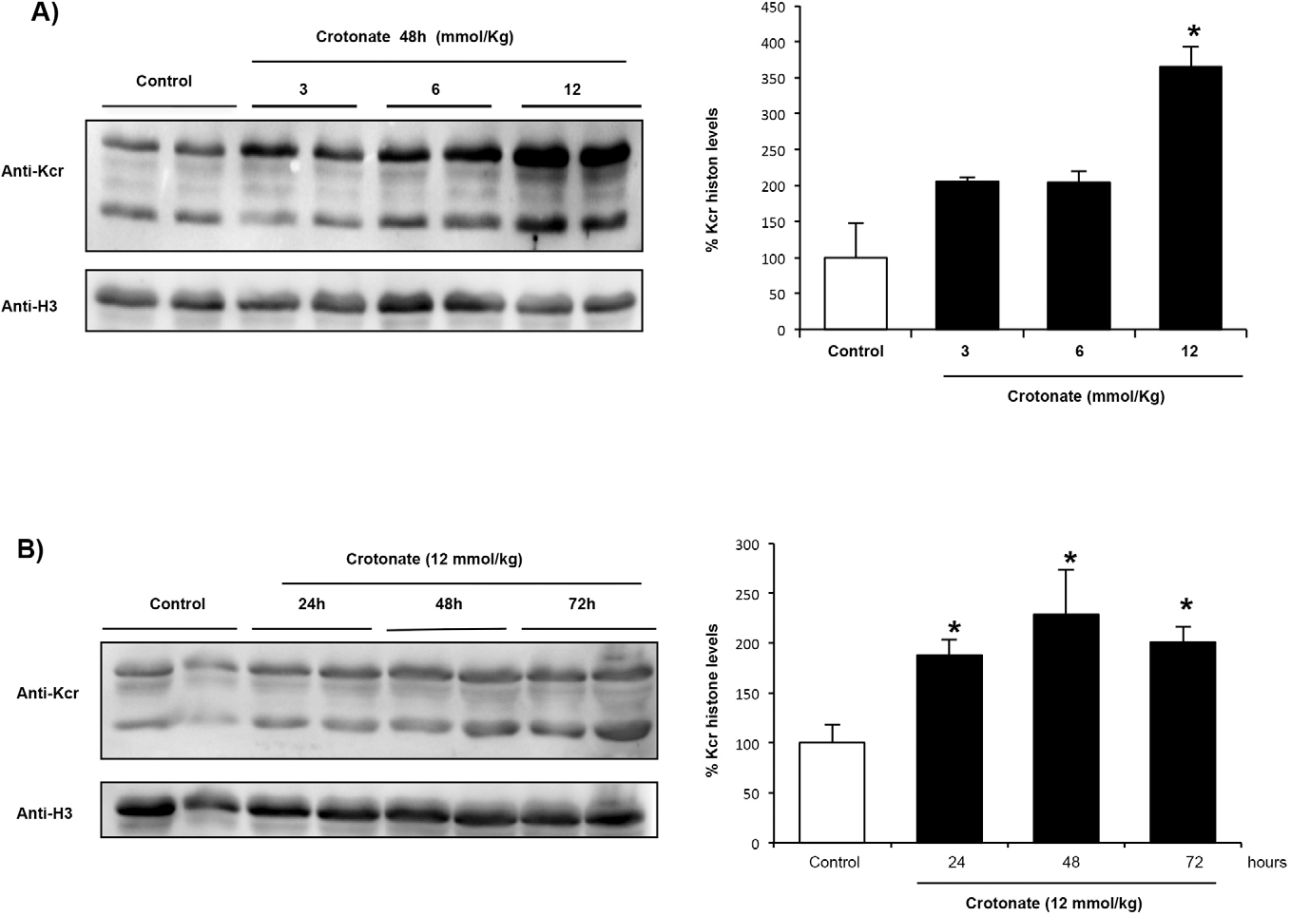
**Crotonate increases histone crotonylation in mouse kidney.** Mice were treated with crotonate at different doses and for different times. Histone crotonylation was measured by western blotting (anti-Kcr). (A) Dose-response curve at 48 h. Mice received vehicle, or 3, 6 or 12 mmol/kg body weight crotonate by intraperitoneal injection. Mean±s.e.m. of four mice per group. *P<0.05 vs control (non-parametric Mann–Whitney *U*-test). (B) Timecourse curve. Mice were killed 24, 48 or 72 h following intraperitoneal injection injection of 12 mmol/kg body weight crotonate or vehicle (control). Mean±s.e.m. of four mice per group. *P<0.05 vs control (non-parametric Mann–Whitney *U*-test).

**Fig. 5.**
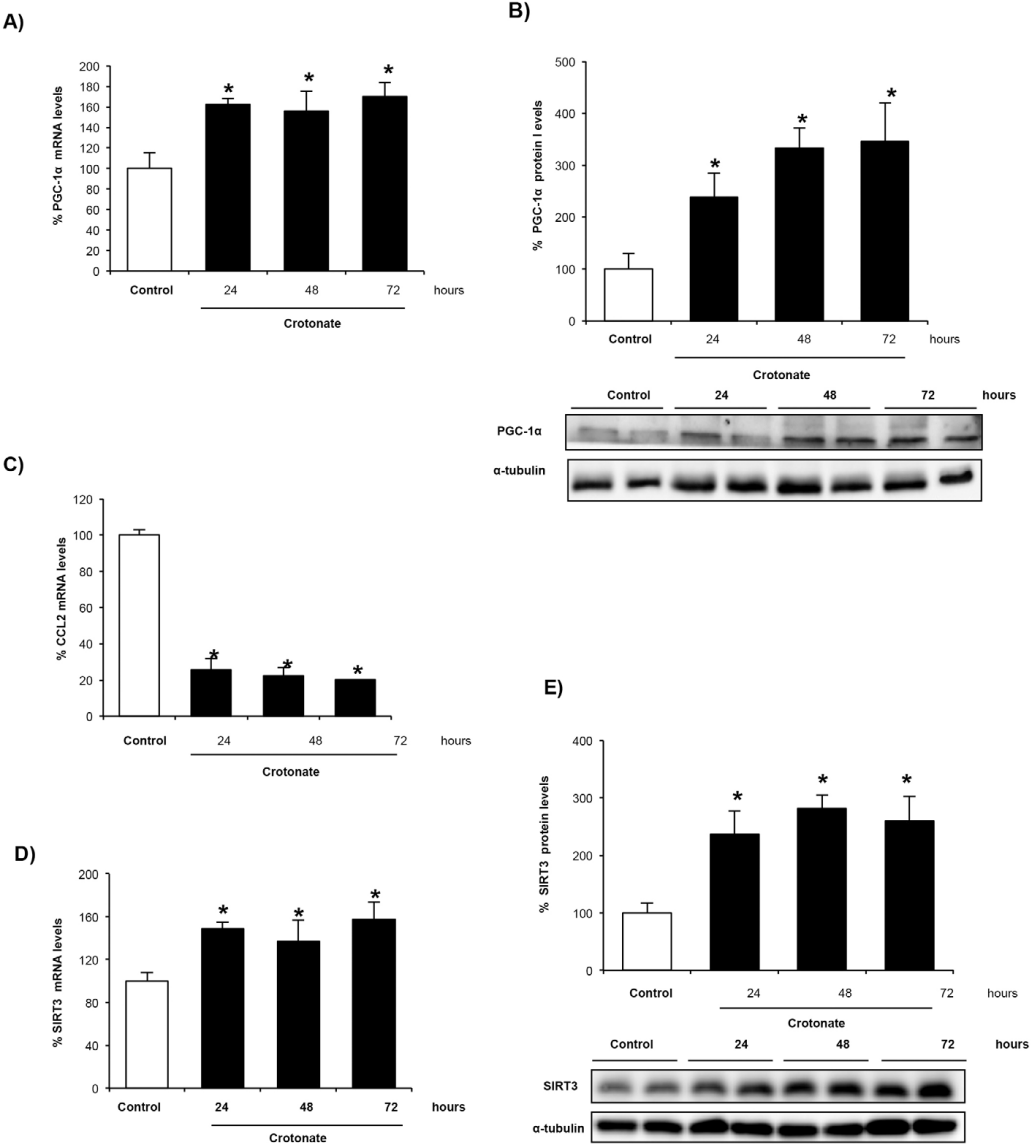
**Crotonate increases PGC-1α and SIRT3 mRNA and decreases CCL2 mRNA expression in mouse kidney.** Mice were treated with 12 mmol/kg body weight crotonate for different times. (A,B) Crotonate increases kidney PGC1α expression at mRNA levels as assessed by qRT-PCR (A) and at the protein level by western blotting (B). Mean±s.e.m. of four per group. *P<0.05 vs control (non-parametric Mann–Whitney *U*-test). (C) Crotonate decreases whole kidney CCL2 mRNA levels as assessed by qRT-PCR. Mean±s.e.m. of four per group. *P<0.05 vs control (non-parametric Mann–Whitney *U*-test). (D,E) Crotonate increases whole kidney SIRT3 mRNA levels as assessed by qRT-PCR (D) and the SIRT3 protein level as assessed by western blotting (E). Mean±s.e.m. of four per group. *P<0.05 vs control (non-parametric Mann–Whitney *U*-test).

We explored whether crotonate modulates kidney histone crotonylation *in vivo*. Systemic administration of crotonate increased histone crotonylation in mouse kidney in a dose- and time-dependent manner (Fig. 4A,B). The 6 mmol/kg body weight crotonate dose did not significantly change kidney histone crotonylation (Fig. S6A) or PGC-1α mRNA expression (Fig. S6B) at 24 h. Thus, 12 mmol/kg body weight crotonate was used for further experiments and was found to increase whole kidney histone crotonylation (Fig. 4), PGC-1α mRNA levels (Fig. 5A) and PGC-1α protein (Fig. 5B), and to decrease kidney CCL2 mRNA levels (Fig. 5C). Thus, the potential nephroprotective actions of crotonate observed in cultured tubular cells (increased expression of the nephroprotective gene PGC-1α and decreased inflammatory gene expression) was reproduced *in vivo*.

Given that the SIRT3 decrotonylase (Bao et al., 2014) and PGC-1α each regulate each other under physiological and stress conditions (Shi et al., 2005; Palacios et al., 2009; Than et al., 2011; Giralt et al., 2011), we studied the effect of exogenous crotonate on kidney SIRT3 expression *in vivo* and found that crotonate increased whole kidney SIRT3 mRNA (Fig. 5D) and protein levels (Fig. 5E). This is consistent with the effects observed in cultured tubular cells and is again suggestive of activation of a negative-feedback loop.

### Crotonate protects from experimental AKI

**Fig. 6.**
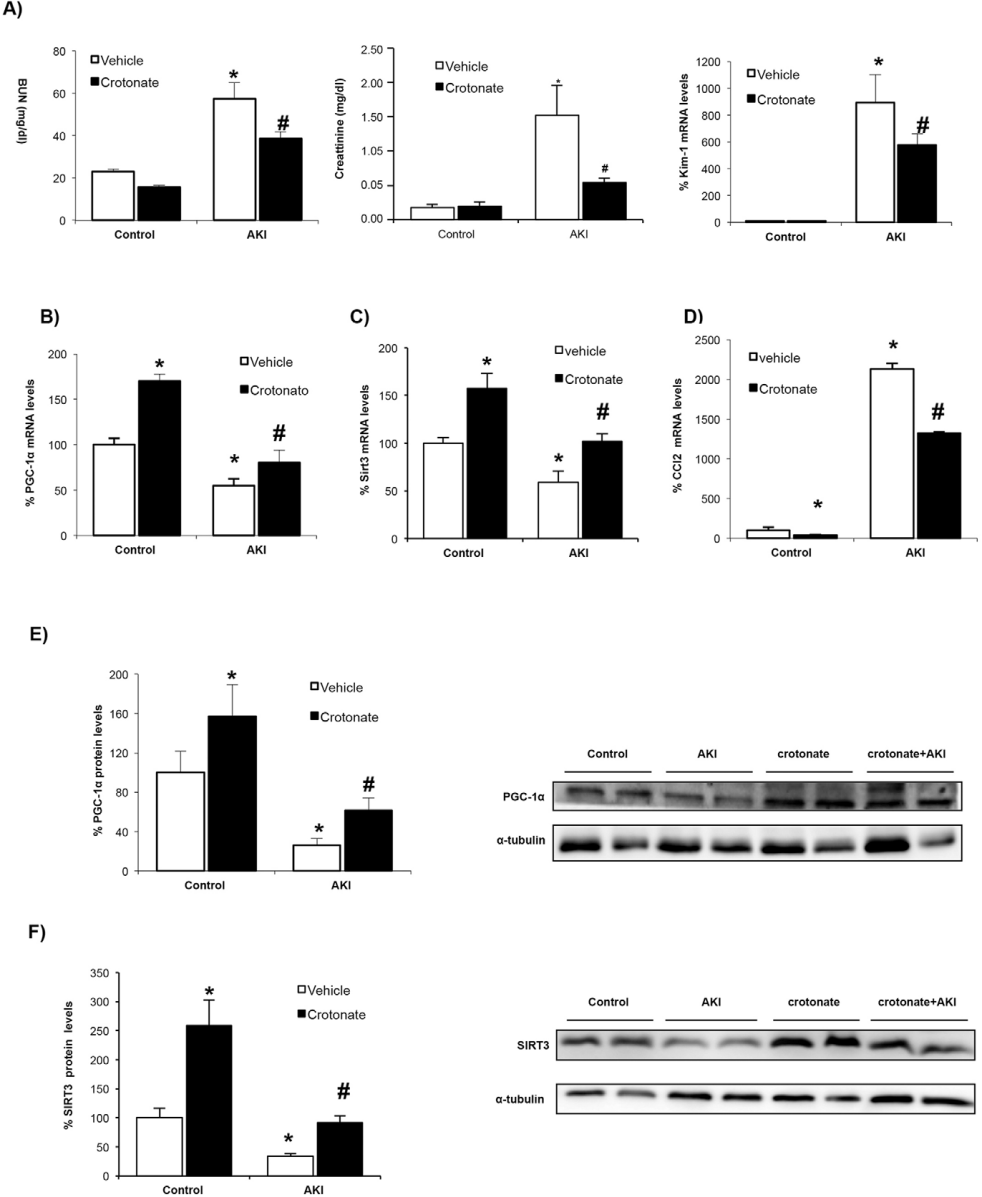
**Crotonate prevents downregulation of kidney PGC-1α and SIRT3 as well as causing CCL2 upregulation in experimental AKI.** AKI was induced by a folic acid overdose in mice pretreated with or without 12 mmol/kg body weight crotonate. All mice were killed at 72 h. (A) Crotonate prevented the increase in serum BUN and creatinine levels and in Kim-1 mRNA expression observed in AKI. Mean±s.e.m. of five mice per group. *P<0.05 vs vehicle control, ^#^*P*<0.05 vs vehicle AKI (non-parametric Mann–Whitney *U*-test). (B-D) The kidney PGC-1α mRNA decrease (B), SIRT3 mRNA decrease (C), and CCL2 mRNA increase (D) were prevented by pretreatment with crotonate. Mean±s.e.m. of five animals per group. *P<0.05 vs vehicle control, ^#^*P*<0.05 vs vehicle AKI (non-parametric Mann–Whitney *U*-test). (E,F) Kidney PGC-1α (E) and SIRT3 (F) protein levels decreased in AKI and this was prevented by pretreatment with crotonate. Western blot of whole kidney protein. Mean±s.e.m. of five animals per group. *P<0.05 vs vehicle control, ^#^*P*<0.05 vs vehicle AKI (non-parametric Mann–Whitney *U*-test).

We next explored whether crotonate was nephroprotective *in vivo*. Mice were pretreated with 12 mmol/kg body weight crotonate, and 24 h later, AKI was induced by a folic acid overdose and mice were killed at 72 h, when renal failure peaks (Sanz et al., 2010b). First, we observed that crotonate resulted in lower serum levels of BUN and creatinine, markers of renal dysfunction severity, and in lower KIM-1 mRNA levels, a marker of kidney injury (Fig. 6A). PAS-stained kidney sections revealed a trend towards decreased tubular injury in crotonate-treated mice (Fig. S7). AKI was associated with increased CCL2 expression (Sanz et al., 2010b) and reduced whole kidney SIRT3 expression (Fig. S8) within the time points studied. In this line, systemic administration of crotonate prevented the decrease in kidney PGC-1α and SIRT3 levels in AKI (Fig. 6B,C,E,F) as well as the increase in CCL2 mRNA expression (Fig. 6D). This suggests a protective effect of crotonate, and thereby of histone crotonylation, against inflammation and mitochondrial stress during AKI.

### TWEAK downregulates SIRT3 and this is prevented by crotonate

**Fig. 7.**
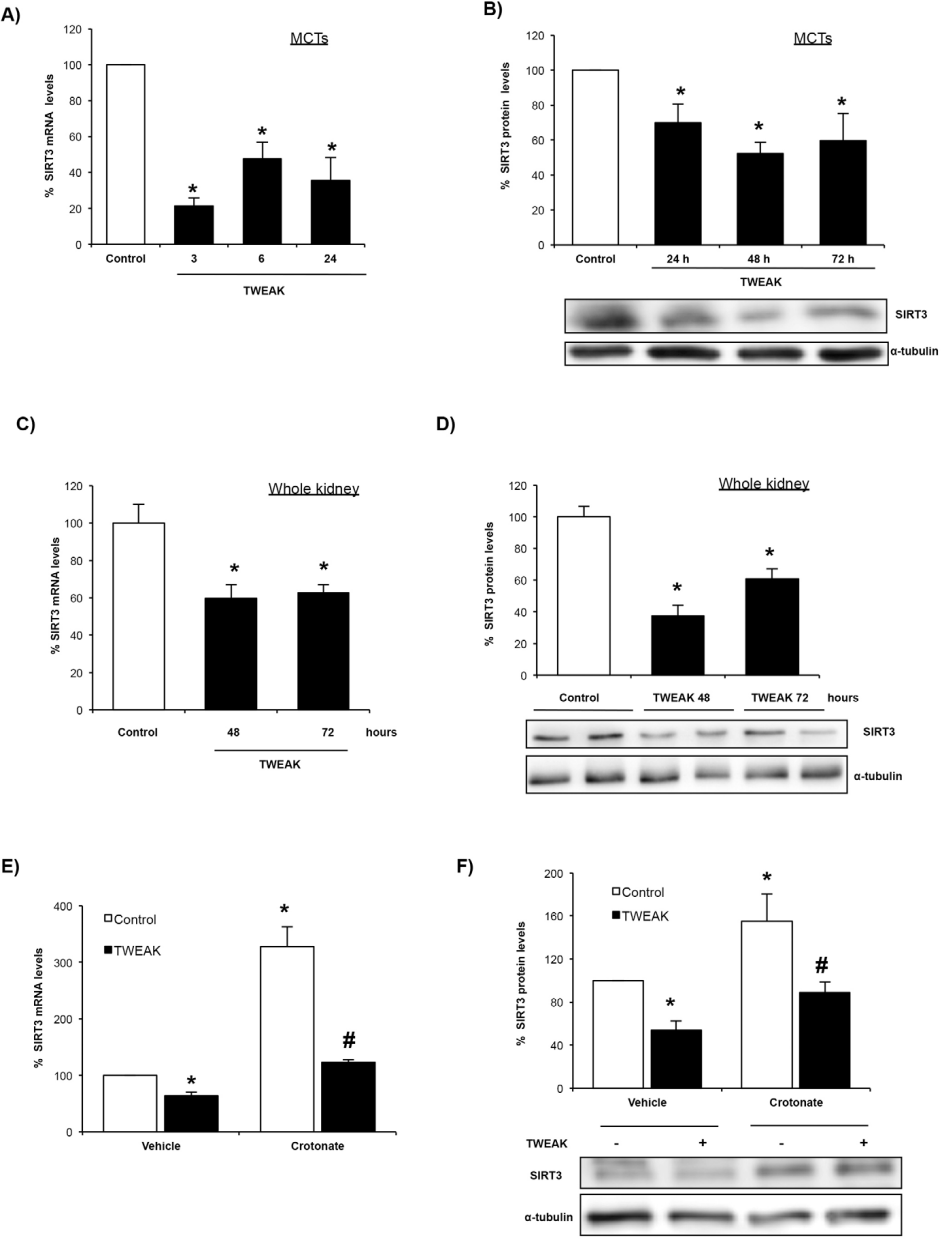
**TWEAK downregulates SIRT3 in cultured tubular cells and this is prevented by crotonate.** (A) SIRT3 mRNA levels in tubular cells incubated with 100 ng/ml TWEAK. Mean±s.e.m. of three independent experiments. *P<0.05 vs control (non-parametric Mann–Whitney *U*-test). (B) Total protein levels of SIRT3 in MCT cells incubated with 100 ng/ml TWEAK for different periods of time. Mean±s.e.m. of three independent experiments. *P<0.05 vs control (non-parametric Mann–Whitney *U*-test). (C) Kidney SIRT3 mRNA levels in mice treated with TWEAK. Mean±s.e.m. of five animals per group. *P<0.05 vs control (non-parametric Mann–Whitney *U*-test). (D) Kidney SIRT3 protein levels in mice treated with TWEAK for different times. Mean±s.e.m. of five animals per group. *P<0.05 vs control (non-parametric Mann–Whitney *U*-test). (E) SIRT3 mRNA levels were tested in tubular cells incubated with 100 ng/ml TWEAK with or without 50 mM crotonate for 6 h. Data from three independent experiments and expressed as mean±s.e.m. *P<0.05 vs vehicle control; ^#^*P*<0.05 vs vehicle TWEAK (non-parametric Mann–Whitney *U*-test). (F) Total SIRT3 protein levels in tubular cells incubated with 100 ng/ml TWEAK with or without 50 mM crotonate for 48 h. Data from four independent experiments and expressed as mean±s.e.m. *P<0.05 vs vehicle control, #*P*<0.05 vs vehicle TWEAK (non-parametric Mann–Whitney *U*-test).

As recently described, increased histone crotonylation in response to crotonate loading might depend on increased substrate (crotonyl-CoA) availability (Sabari et al., 2015). However, the mechanism of increased histone crotonylation following TWEAK stimulation remained unclear. Thus, we explored whether TWEAK regulated the expression of the SIRT3 decrotonylase and observed that TWEAK downregulated SIRT3 at mRNA and protein levels in cultured tubular cells (Fig. 7A,B) and in whole kidney *in vivo* (Fig. 7C,D). TWEAK-induced SIRT3 downregulation was prevented by crotonate in cultured tubular cells (Fig. 7E,F). These data suggest that decreased SIRT3 expression might be one of the factors contributing to increased kidney cell histone crotonylation in response to TWEAK.

## DISCUSSION

The main findings of this study are that the degree of histone crotonylation in kidney tubular cells is modified by certain cell stressors or crotonate. Furthermore, increasing histone crotonylation was beneficial overall in AKI. This is the first observation of the *in vivo* potential of the therapeutic manipulation of histone crotonylation in a disease state.

Histones were the most abundant crotonylated proteins. The fact that increased histone crotonylation was found under stress conditions, be it AKI or exposure to a proinflammatory cytokine, begs the question of what is the overall role of crotonylation in kidney injury. As is the case with other histone post-translational modifications, it is expected that, in response to the microenvironment, expression of some genes will increase whereas that of other genes will decrease depending on the degree of histone crotonylation. Despite this expected heterogeneity, therapeutic agents targeting other histone post-translational modifications have been beneficial in diverse pathological conditions, including kidney injury, even when potentially impacting the expression of multiple genes with diverse or even opposing functions. For example, HDAC inhibitors such as trichostatin A are protective in experimental models of kidney fibrosis (Van Beneden et al., 2013) and selectively mitigate the stimulatory effect of lipopolysaccharide on inflammatory cytokine expression (Munro et al., 2013). We hypothesized that overall interference with histone crotonylation might have a beneficial or deleterious effect in AKI. We have now shown that increasing overall histone crotonylation by exposure to crotonate has potentially beneficial effects on tubular cells in culture and *in vivo*, including an increased expression of the mitochondrial biogenesis regulator PGC-1α and decreased chemokine expression. Consistent with these findings, crotonate protected from injury and loss of renal function in AKI. The genes studied in the present manuscript were chosen as representative of upregulated and downregulated genes shared by both AKI and stressor-stimulated (i.e. TWEAK-stimulated) cultured tubular cells. Given that histone post-translational modifications might impact on the expression of multiple genes, it remains to be explored whether changes in the expression of these specific genes or other genes are the key drivers of the observed beneficial effect of crotonate.

We identified and characterized two interventions that increased overall histone crotonylation in kidney cells: cell stress by inflammatory cytokines or during AKI, and increasing the crotonate substrate availability. By contrast, direct cytotoxicity mediated by cisplatin in culture did not modulate histone crotonylation. However, both interventions had a differential effect on the expression of the studied genes. Thus, we identified two specific genes, PGC-1α and SIRT3, which underwent increased histone crotonylation during AKI and in tubular cells stressed by TWEAK. Under stress conditions, the mRNA and protein levels of both genes were decreased, suggesting decreased transcription. One possible explanation is that increased histone crotonylation results in decreased gene transcription. However, increasing overall histone crotonylation by addition of crotonate increased the expression of PGC-1α and SIRT3. Thus, alternative explanations should be sought. One possibility is that the effect of histone crotonylation might be dependent on the context: within the increased crotonate substrate availability context, histone crotonylation could promote gene expression whereas under a proinflammatory or cell stress context, histone crotonylation might decrease expression of certain genes. In this regard, the same histone post-translational modification might be associated with increased or decreased gene expression depending on the gene and context (Bao et al., 2014; Sabari et al., 2015). Further studies should clarify this issue in the specific case of crotonylation. Up to now, higher levels of histone crotonylation at the promoters of genes activated by lipopolysaccharide, such as Il6, Gbp2, Ifit1 and Rsad2, have been associated with increased gene expression (Sabari et al., 2015). In this regard, an alternative potential explanation is that increased histone crotonylation at the genes encoding SIRT3 and PGC-1α following cell stress might be a compensatory mechanism that limits the fall in gene expression, rather than the driver of gene suppression. Further studies are needed to unravel the role of histone crotonylation in the regulation of gene expression in different cellular contexts and for specific genes.

It has recently been reported that crotonate increases the intracellular crotonyl-CoA availability, thus stimulating gene transcription through p300 (also known as EP300)-catalyzed histone crotonylation (Sabari et al., 2015). In this regard, histone crotonylation can be catalyzed by either p300 or the p300-CREB-binding protein (CBP) complex (Sabari et al., 2015). Interestingly, SIRT3 and PGC-1α regulate each other through CREB-mediated gene expression mechanisms (Shi et al., 2005; Palacios et al., 2009; Than et al., 2011; Giralt et al., 2011). Our results suggests that crotonate, by increasing crotonyl-CoA availability, leads to increased histone crotonylation (Sabari et al., 2015), and increased gene transcription of SIRT3, that, in turn, acts as a decrotonylase. The increased expression of a decrotonylase in response to crotonate might be teleologically interpreted as the activation of a negative-feedback mechanism. Increased SIRT3 levels and histone crotonylation at the PGC-1α gene might increase PGC-1α levels, limiting cell injury in response to stress.

**Fig. 8.**
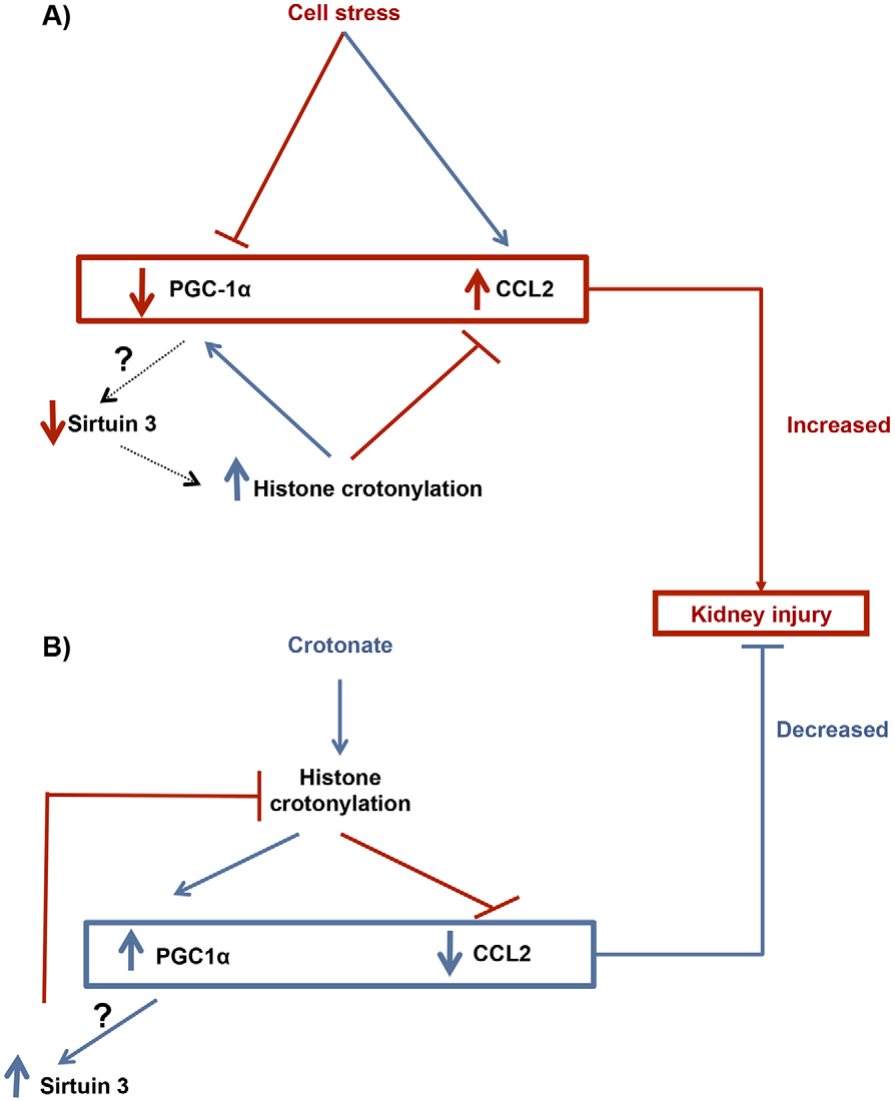
**Working hypothesis of histone lysine crotonylation in kidney injury.** (A) Kidney cell stressors. Cell stressors, such as TWEAK, decrease PGC-1α and increase CCL2 expression. These changes might contribute to tissue injury. We hypothesize that the decreased PGC-1α expression might contribute to decreased expression of the crotonylase SIRT3 and, this, in turn, limits the decrease in PGC-1α and SIRT3 expression by promoting histone crotonylation at the PGC-1α and SIRT3 genes, as observed in cultured cells. (B) Therapeutic response to crotonate. Crotonate increased overall histone crotonylation and increased the expression of PGC-1α and SIRT3, and decreased CCL2 expression. We hypothesize that these changes might contribute to the observed nephroprotection afforded by crotonate. The increased SIRT3 expression could, in turn, limit histone crotonylation as a negative-feedback mechanism.

By contrast, under stress conditions, such as AKI or in tubular cells stressed by inflammatory cytokines (e.g. TWEAK), the decrease in SIRT3 expression, and, potentially, in SIRT3 decrotonylase activity, might lead to increased histone crotonylation, thus limiting the downregulation of protective genes such as PGC-1α (Fig. 8). Thus, decreased SIRT3 expression might be an additional pathway leading to increased histone crotonylation in an inflammatory milieu. Further research should explore the changes of histone crotonylation in relation to the functional status of the cell, as well as identify changes in histone crotonylation for individual genes.

SIRT3 is the physiological deacetylase that antagonizes p300-mediated histone acetylation (Wang et al., 2012). Although sirtuins were initially described as NAD-dependent deacetylases (Imai et al., 2000; Landry et al., 2000; Sauve et al., 2006), some sirtuins with weak deacetylase activity might have substrate specificity towards other acyl groups attached to lysine residues. For example, SIRT5 preferentially hydrolyzes malonyl and succinyl lysine (Jiang et al., 2013; Du et al., 2011; Peng et al., 2011), and SIRT6 can remove long chain fatty acyl groups from lysine residues (Jiang et al., 2013). More recently, SIRT1 to SIRT3 have been reported to behave as decrotonylases, thus regulating histone crotonylation dynamics and gene transcription (Bao et al., 2014).

A staining pattern compatible with histone crotonylation was also observed in human kidney tissue, including diseased kidneys. Although we were not able to study early stages of human AKI, this observation supports the potential clinical relevance of the findings.

In conclusion, for the first time we have shown that the pattern of histone crotonylation changes during AKI and in cultured tubular cells stressed by an inflammatory cytokine, suggesting a role of histone crotonylation in kidney injury. Furthermore, we have shown that the degree of kidney cell histone crotonylation might be manipulated therapeutically by administering crotonate, and that increasing overall histone crotonylation is nephroprotective. Although the precise mechanisms for the protective effect of crotonate remains to be further clarified, results reported here are consistent with the hypothesis that histone-crotonylation-mediated regulation of gene transcription plays a role. This is the first observation of the *in vivo* potential of the therapeutic manipulation of histone crotonylation in a disease state. Further studies are needed to define the role of histone crotonylation in additional models of kidney disease and in injury to other tissues.

## MATERIALS AND METHODS

### Cells and reagents

For *in vitro* experiments we used MCT murine proximal tubular epithelial cells. This cell line was originated from the kidneys of SJL mice in the University of Pennsylvania and obtained from Eric G. Neilson. Cells were authenticated and tested for mycoplasma contamination before use. MCT cells were cultured in RPMI 1640 with 10% fetal bovine serum (FBS), 2 mM glutamine and antibiotics (100 U/ml penicillin and 100 µg/ml streptomycin), in 5% CO_2_ at 37°C. RPMI-1640, penicillin, streptomycin and trypsin-EDTA were from BioWhittaker (Waltham, MA) and FBS from Gibco (Carlsbad, CA) (Haverty et al., 1988). For experiments, cells were serum-depleted for 24 h, and then stimulated. Recombinant human TWEAK (Millipore, Billerica, MA) was used at 100 ng/ml unless otherwise specified, based on previously reported dose-response experiments in the same cells (Sanz et al., 2008b). Crotonate was used at 50 mM and 100 mM, based on previous studies on dynamics of histone lysine crotonylation in response to crotonate (Tan et al., 2011). Crotonic acid (Sigma) was dissolved in water at a concentration of 100 mg/ml to yield a clear, colorless solution. A 1 M stock solution in double-distilled H_2_O was adjusted to neutral pH (pH 7.5) with sodium chloride. From this stock solution, the necessary dilutions were made in cell culture medium (RPMI-1640, Sigma). Following the same protocol, we used mannitol (Sigma), at concentrations equimolar to the crotonate concentrations used, as an osmolarity control in experiments *in vitro*. Mannitol is an inert molecule generally used as a osmolarity control for experimental conditions that increase osmolarity (e.g. during the study of the effects of high glucose concentration on cell biology). Cisplatin (Sigma) was used at concentrations of 50 and 100 µM.

### RNA extraction and real-time PCR

Total RNA was extracted by the TRI Reagent method (Sigma) and 1 µg of RNA was reverse transcribed with the high capacity cDNA archive kit (Applied Biosystems, Foster City, CA). TaqMan Gene Expression Assays were from Applied Biosystems. Quantitative PCR was performed in a 7500 Real Time PCR System with the Prism 7000 System SDS Software (Applied Biosystems) and RNA expression was corrected to the GAPDH expression.

### Western blotting

Cell samples were homogenized in lysis buffer (50 mM Tris-HCl, 150 mM NaCl, 2 mM EDTA, 2 mM EGTA, 0.2% Triton X-100, 0.3% NP-40, 0.1 mM PMSF and 1 µg/ml pepstatin A) then separated by 10% SDS-PAGE under reducing conditions. After electrophoresis, samples were transferred to nitrocellulose membranes (Bio-Rad), blocked with 5% skimmed milk in PBS with 0.5% (v/v) Tween 20 for 1 h, washed with PBS with Tween, and incubated with mouse polyclonal anti-PGC-1α (1:1000, ST1202, Calbiochem) or rabbit polyclonal anti-SIRT3 (1:1000, #5490, Cell Signaling). Anti-PGC-1α was diluted in 5% milk PBS with Tween and anti-SIRT3 in 5% BSA in PBS with Tween. Blots were washed with PBS containing Tween and incubated with the appropriate horseradish peroxidase (HRP)-conjugated secondary antibody (1:2000, Amersham, Aylesbury, UK). After washing with PBS containing Tween, blots were developed with the chemiluminescence method (ECL) (Amersham) using an ImageQuant LAS 400 system (GE Healthcare). Then, the images were analyzed with Quantity One software (Bio-Rad). Blots were then probed with mouse monoclonal anti-α-tubulin antibody (1:10,000, Sigma) and the levels of expression were corrected for differences in loading.

Histones were isolated using the epiQuik Total histone Extraction kit (Epigentek), and then separated by 10% SDS-PAGE under reducing conditions. After electrophoresis, samples were transferred to PVDF membranes (Millipore), blocked with 5% BSA in PBS with 0.5% (v/v) Tween 20 for 1 h, washed with PBS containing Tween, and incubated with rabbit polyclonal pan anti-crotonyl-lysine antibody (1:1000, PTM-501, PTM Biolabs) and anti-crotonyl-Histone H3 (Lys9) antibody (anti-H3K9, 1:1000, PTM-516, PTM Biolabs). Both antibodies were diluted in 5% BSA in PBS with Tween. Blots were then probed with rabbit polyclonal anti-histone H3 antibody (1:2000, #9715, Cell Signaling) after stripping with a buffer that removes primary and secondary antibodies from membranes (Yeung and Stanley, 2009). Anti-Kcr staining was normalized to anti-Histone H3 or Ponceau staining for each sample to correct for differences in loading. Then, the mean of the normalized control values was set at 100% and the normalized expression levels in other samples was expressed as a percentage change over control.

### Animal models

All procedures were conducted in accordance with the NIH Guide for the Care and Use of Laboratory Animals and were approved by the Animal Ethics Committee of IIS-FJD. Folic acid nephropathy is a classical model of AKI (Sanz et al., 2008a; Ortega et al., 2006; Fang et al., 2005; Doi et al., 2006), which has been described in humans (Metz-Kurschel et al., 1990). Mice received a single intraperitoneal injection of 250 mg/kg body weight folic acid (Sigma) in 0.3 mol/l sodium bicarbonate or vehicle control and were killed at days 1, 3 or 7 (*n*=5 per day and group).

As a second model of AKI another set of mice (*n*=5 per group) was injected intraperitoneally with 20 mg/kg body weight cisplatin (Sigma) or vehicle (saline control) (Zhang et al., 2008). Mice were killed at 72 h. Nephrotoxicity is the dose-limiting side effect of the chemotherapeutic agent cisplatin in humans.

A further set of mice was injected intraperitoneally with crotonate. For dose-response experiments, mice received 3, 6 or 12 mmol/kg body weight crotonate (Sigma) or vehicle (saline control) and were killed at 48 h. For timecourse experiments, mice were injected intraperitoneally with 12 mmol/kg body weight crotonate or vehicle (saline control) (Hawkins et al., 1973; Lemieux et al., 1979; Lemieux et al., 1977) and killed at 24, 48 or 72 h (*n*=4 per group and time-point). Crotonate dose was calculated based on *in vitro* experiments for an extracellular volume of 6.5 ml/mouse and further refined by the dose-response studies. In another set of experiments, mice were pretreated with 12 mmol/kg body weight crotonate and 24 h later AKI was induced by folic acid injection and mice were killed at 72 h.

Kidneys were perfused *in situ* with cold saline before removal. One kidney was snap-frozen in liquid nitrogen for RNA and protein studies, and the other was fixed and embedded in paraffin. Blood was collected from the femoral vein before perfusion of the kidneys. For all the experiments, C57/BL6 mice of 12 to 14 weeks old were used.

For histological assessment, PAS-stained kidney sections were evaluated by an experienced pathologist blinded as to the nature of the samples, using a semiquantitative histological score on a 0 (normal) to 3 scale (severely affected) evaluating the following items: tubular cell injury, tubular cell regeneration, tubular atrophy, calcification, tubule dilatation, leukocyte casts and hyaline casts. For each mouse, the sum of the individual score for each item yielded the total score.

### ChIP-seq

ChIP-seq for histone lysine crotonylation was carried out as previously described with 100 µg fractionated cell or kidney tissue chromatin and 5 µg anti-crotonyl-lysine antibody (Tan et al., 2011). ChIP-seq libraries for sequencing were prepared following Illumina protocols (Illumina, San Diego, CA) with minor modifications. Libraries for input samples were generated using 20 ng of corresponding input chromatin. Briefly, ChIPed DNA was first blunted with an END-IT DNA repair kit (Epicenter Biotechnology, Madison, WI) and then incubated with Klenow fragment (3′→5′ exo) (New England Biolabs, MA) and dATP to generate a single-base 3′-dA overhang. Illumina sequencing adapter was then ligated to the resulting DNA, followed by size selection (180–400 bp) on an 8% acrylamide gel. This size-selection step was repeated after PCR amplification with DNA primers (Illumina). Libraries were sequenced using Illumina GAII or HiSeq machine as per the manufacturer's protocols. Following sequencing cluster imaging, base calling was conducted using the Illumina pipeline. Reads were mapped to the mouse mm10 genome build with a bowtie software package. Total mapped tags were paired down to unique, monoclonal tags. These are tags that mapped to one location in the genome and each sequence is represented once.

### Flow cytometry

A total of 10,000 cells were seeded in 12-well plates (Costar, Cambridge, MA) in RPMI with 10% FBS overnight and rested in serum-free medium for 24 h before crotonate addition. Thereafter, stimuli were added to subconfluent cells. For assessment of apoptosis by flow cytometry adherent cells were pooled with spontaneously detached cells, and incubated in 100 µg/ml propidium iodide, 0.05% NP-40 and 10 µg/ml RNAse A in PBS at 4°C for >1 h. This assay permeabilizes the cells, thus the propidium iodide stains both living and dead cells. The percentage of apoptotic cells with decreased DNA staining (hypodiploid cells) was assessed by counting after flow cytometry using BD CellQuest Software (BD Biosciences) (Sanz et al., 2009; Justo et al., 2006; Lorz et al., 2000).

### Immunohistochemistry and immunofluorescence

Kidney tissue immunohistochemistry was performed as previously described (Hazzouri et al., 2000) in 3-µm thick sections of paraffin-embedded tissue using a PT-link device (with a low pH solution, 95°C, 20 min). Sections were washed with wash buffer for 5 min and blocked by incubation with PBS containing 5% milk for 30 min. For immunostaining, sections were incubated with anti-crotonyl-lysine antibody (PTM Biolabs) diluted at 1:250 in TBS 0.5% milk for 2 h, washed in TBS three times, incubated with biotinylated secondary antibody (1:2000 in PBS containing 0.5% milk) for 30 min, and washed and incubated with AB streptavidin complex. Then the slides were washed with TBS and final detection was performed using DAB (Dako Diagnostics) according to the manufacturer's instructions. Sections were counterstained with Carazzi's hematoxylin. Negative controls included incubation with isotype IgG. Immunohistochemistry was performed in five human kidney samples from the IIS-FJD Biobank, corresponding to males, aged 56 to 80 years, serum creatinine 0.7 to 1.7 mg/dl. Informed consent was obtained for all tissue donors and all clinical investigations have been conducted according to principles expressed in the Declaration of Helsinki.

For immunofluorescence, 10^6^ cells were seeded on coverslips in RPMI medium supplemented with 10% FBS and were serum starved for 24 h prior to experiments. Cells were washed with ice-cold PBS three times and fixed in neutral buffered 10% formalin (Sigma) in PBS for 20 min at room temperature. After three brief PBS rinses, cells were permeabilized with 0.2% Triton X-100 in PBS for 10 min on ice followed by PBS rinses. Permeabilized cells were then blocked with 5% BSA in PBS for 30 min at room temperature and then incubated with pan anti-crotonyl-lysine antibody (PTM Biolabs) at 1:500 in PBS with 5% BSA at 4°C overnight, followed by incubation with Alexa-Fluor-488-conjugated goat anti-rabbit-IgG (1:300 in PBS with 5% BSA, Invitrogen) for 1 h at 37°C. Then cells were incubated with Alexa-Fluor-555–phalloidin (1:1500 in PBS with 5% BSA; Life Technologies) for 30 min at room temperature, counterstained with DAPI and mounted.

### Statistics

Statistical analysis was performed using the SPSS 11.0 statistical software (Chicago, IL). Results are expressed as mean±s.e.m. Significant differences between mean values were determined with the Mann–Whitney *U*-test for comparison of two groups or paired Student's *t*-test if appropriate (normal distribution and *n*≥5). Two-tailed test values of *P*<0.05 were considered significant. For small samples sizes (*n*<5), a nonparametric Mann–Whitney *U*-test was assessed.
